# Trends of central obesity and associations with nutrients intake and daily behaviors among women of childbearing age in China

**DOI:** 10.1186/s12905-022-01600-9

**Published:** 2022-01-12

**Authors:** Zichong Long, Lili Huang, Jiajun Lyu, Yuanqing Xia, Yiting Chen, Rong Li, Yanlin Wang, Shenghui Li

**Affiliations:** 1 School of Public Health, Shanghai Jiao Tong University School of Medicine, 227 South Chongqing Road, Huangpu District, 200025 Shanghai, China; 2 Prenatal Diagnosis Department, International Peace Maternity and Child Health Hospital, Shanghai Jiao Tong University School of Medicine, 910 Hengshan Road, Xuhui District, 200030 Shanghai, China; 3 MOE - Shanghai Key Laboratory of Children’s Environmental Health, Xinhua Hospital, School of Medicine, Shanghai Jiao Tong University, Shanghai, China

**Keywords:** Women of childbearing age, Central obesity, Physical activity, Nutrients

## Abstract

**Background:**

Obesity among women of childbearing age has becoming an important public health concern. We aimed to describe the trends of central obesity among Chinese women of childbearing age from 2004 to 2011 and to examine its associations with nutrients intake and daily behaviors.

**Methods:**

Longitudinal data were derived from the China Health and Nutrition Survey. Participants consisted of 2481 women aged 15–44 years old. WC (Waist circumference) and WHtR (Waist to height ratio) were adopted as indicators of central obesity. Generalized linear mixed model was performed to analyze the associations of nutrients intake and daily behaviors with central obesity.

**Results:**

From 2004 to 2011, the prevalence of central obesity among Chinese women of childbearing age increased from 21.6 to 30.7% (WC as indice) or from 22.8 to 32.6% (WHtR as indice) (both *p* < 0.001). Protein intake above the AMDR (Acceptable macronutrient distribution range) (OR = 1.21, 95% CI 1.05–1.39, *p* < 0.01) and non-participation in LTPA (Leisure time physical activity) (OR = 1.45, 95% CI 1.17–1.80, *p* < 0.001) were risk factors for high WC, and the latter was also associated with high WHtR (OR = 1.36, 95% CI 1.10–1.67, *p* < 0.01). For those women who had high WC & high WHtR, the impacts of protein intake and LTPA became stronger, especial LTPA (OR = 1.53, 95% CI 1.21–1.94, *p* < 0.001). Age-stratified analyses found that non-participation in LTPA was key factor for central obesity in 15–34 age group, while protein intake above the AMDR was pronounced in the 35–44 age group.

**Conclusions:**

Non-participation in LTPA and protein intake above the AMDR were significant contributors of central obesity, which could be intervention targets to deal with the growing trend of central obesity among women of childbearing age.

**Supplementary Information:**

The online version contains supplementary material available at 10.1186/s12905-022-01600-9.

## Background

Obesity has been a pervasive concern all around the world across different groups of people and all ages. As a particular group, childbearing age women’s obesity is receiving intensive attention [1]. In 2011–2012, the prevalence of overweight/obesity among women aged 20–39 years in the US was 58.5% [2]. In China, a survey conducted in rural areas presented that the prevalence of overweight/obesity among women of 20–49 years old was 24.8% during the period of 2011–2014 [3], another literature indicated that for Chinese women of 15–49 year old, the prevalence of overweight increased from 32.1 to 43.1%, and the prevalence of obesity increased from 5.3 to 10.0% from 2004 to 2015 [4].

The impact of obesity on women of childbearing age is multifaceted and has its specificities. It has been confirmed that excessive weight gain among women of childbearing age is associated with menstrual irregularity, endometrial pathology, and infertility [5]. Furthermore, pre-pregnancy obesity was found to be predictors of a series of complications during pregnancy [6–8]. According to the developmental origins of health and disease theory, childhood health is considered to originate in the periconceptional period [9]. Maternal peri-conceptional weight can alter the intrauterine environment, which may affect the development of the fetus, thus leading to long-term health effects to their offspring. Recently published data has provided evidence that maternal pre-pregnancy obesity was involved in childhood disease susceptibility, including obesity, cognitive or behavioral disorders, and even childhood allergic diseases [10, 11].

Central obesity is characterized by excessive fat deposition in the abdominal region [12]. In recent years, accumulating studies suggested that central obesity indicators, such as WC (waist circumference) and WHtR (waist to height ratio), were more effective to identify obesity-related risks than BMI [13, 14]. Although a large number of genetic and environmental were identified to be associated with energy balance regulation, diets and daily behaviors are still the most primary as well as modifiable factors for obesity [15]. Given the above, women of childbearing age, as a particularly vulnerable people, should be given high priority when discussing obesity. However, to our knowledge, little is known about their obesity prevalence and change trend; also, the long-time tracks of diet and behavior patterns with the change of obesity in women of childbearing age are rarely described and analyzed either. To fill in the existing gaps in the prior literature, in this study, we made a description of the secular trend of central obesity among Chinese women of childbearing age over the duration from 2004 to 2011, and furtherly examined the associations with nutrients intake and daily behaviors.

## Methods

### Study design, setting and subjects

**Fig. 1:**
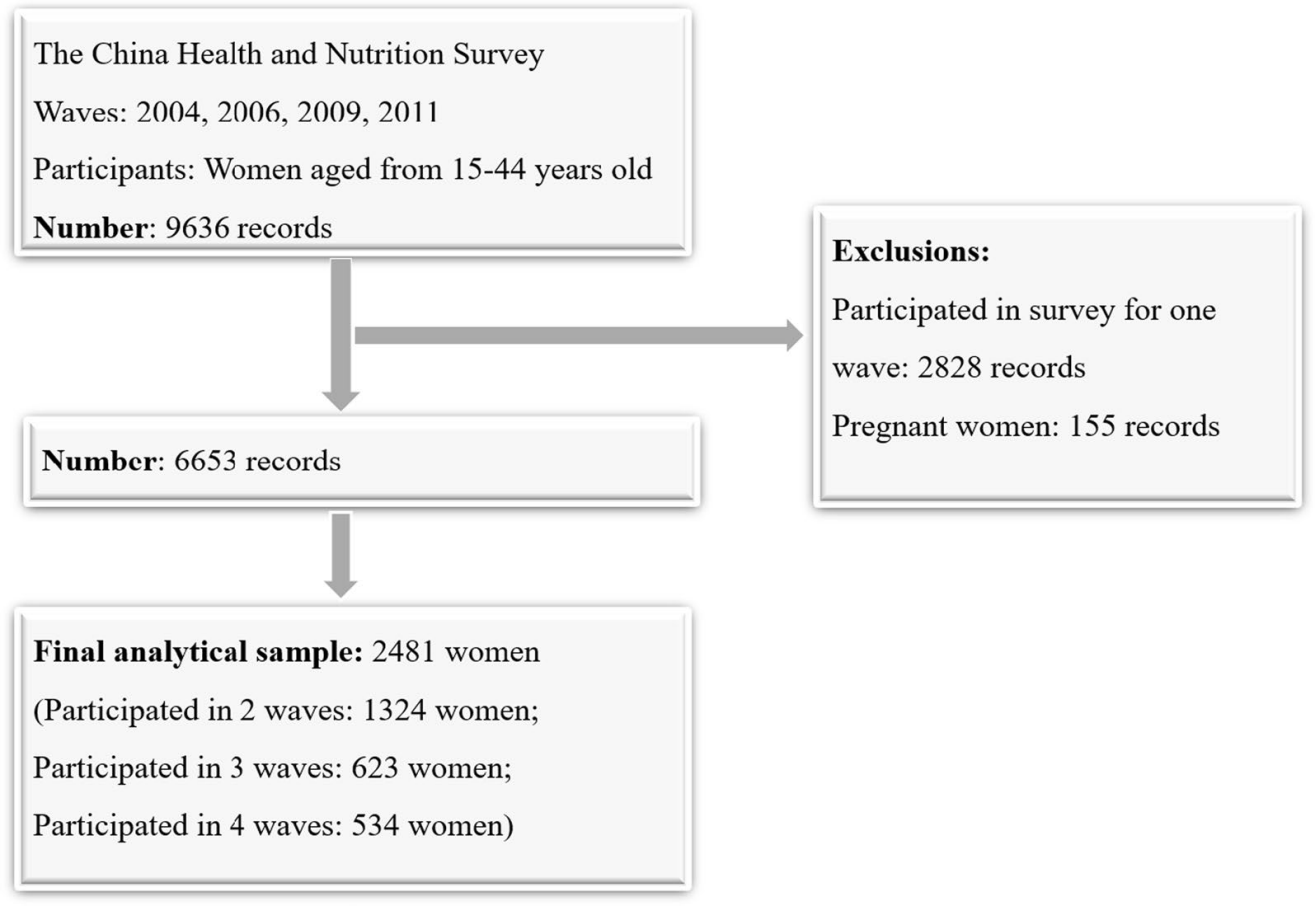
Participants' flow chart

The data of this study was extracted from the China Health and Nutrition Surveys (CHNS), an ongoing longitudinal survey, more details about CHNS were reported elsewhere [16]. In brief, a multistage, random cluster sampling design was carried out from 15 provinces, and then to 2 cities, 4 counties, thirdly to 4 committees in city, 4 villages in county respectively, and finally to 20 households from every committees or villages in sequence. Due to the key analysis variables collected differ from waves, to obtain the most comprehensive energy-balance-related variables, records of four waves (2004, 2006, 2009, 2011) were included in this study. From 2004 to 2011 of 4 waves, a total of 9636 records of women aged 15–44 years were enrolled in the CHNS. After excluding for the women who were pregnant (155 person-years), and participated in survey for only one wave (2828 person-years), 6,653 records consisted of study sample, of which 2481 women were included (1324 women participated in survey for two waves, 623 women for three waves and 534 women for four waves) (Fig. 1). Extreme (mean ± 3 standard deviation) and missing values were excluded from analysis. The age range of women of childbearing age was defined by reference to the recommendation of World Health Organization [17]. The study was approved by the Institutional Review Boards of the University of North Carolina at Chapel Hill, and the Institute of Nutrition and Health, China Center for Disease Control and Prevention.

### Data collection and measurements

Height, weight, and waist circumference were extracted to measure central obesity of participants. In order to check the energy intake and consumption of the objects comprehensively, the intake and energy supply proportion of macronutrients were collected to measure the rationality of energy intake structure; daily activities including LTPA, sedentary behavior and sleep duration were enrolled to reflect energy expenditure of the subjects. Major sociodemographic variables were collected as controlling factors to exam the associations between the above variables and central obesity.

The height and WC of participants were measured by well-trained workers following standard protocols. Height was measured by a portable rangefinder without shoes, and WC was measured at the midpoint between the costal margin and the iliac crest by SECA tape. The measurement of Height and WC were account to the nearest 0.1 cm. WHtR was calculated through WC/height. Previous studies usually adopted unified cut-off value of WC and WHtR to define central obesity [18]. However, since the waist circumference increases with age, several new studies have recommended that the cut-off should take age into account [19, 20]. In this study, all participants were grouped into two age groups as 15–34 age group and 35–44 age group, and ≥ age group-specific P75 of WC (high WC) and WHtR (high WHtR) were used to define central obesity [13, 21].

The data on nutrients intake were collected by three-consecutive 24 h recalls (including two working days and one weekend day). Trained investigators conducted face-to-face interviews to record the types and quantities of food and drinks consumed by each participant, including three meals and snacks intakes, and the ways they cooked meals and the places they had meals. Based on Chinese Food Composition Table, the daily nutrients intake was calculated. Chinese Food Composition Table (2004 version) was applied to 2004 and 2006 surveys, and Chinese Food Composition Table (2009 version) was applied to 2009 and 2011 surveys [22]. According to the Chinese dietary reference intakes-Part 1: Macronutrient (WS/T578.1-2017) (file was downloaded on website: https://www.cnsoc.org/policystand/page2.html), the adequate intake of every macronutrient account for total energy is 50–65% for carbohydrate, 20–30% for fat, and 10–15% for protein, respectively. Based on this standard, we divided the intake level of every macronutrient into < AMDR (below the range), =AMDR (within the range), and > AMDR (above the range).

The CHNS survey defined LTPA for 6 types of physical activity, including martial arts, gymnastics, track and field, football, basketball, volleyball/badminton and others. In our sampled women of childbearing age, nearly 90% of them did not do LTPA, therefore we categorized LTPA into Yes versus No.

Two self-reported questionnaires were applied to evaluate sedentary behaviors in CHNS [1]. Screen exposure questionnaire examined daily time spent on each screen behavior through 8 items, including TV, video tapes, CDs and DVDs, watching movies and videos online, video games, surfing the internet, participating in chat rooms, and playing computer games [2]; Homework and other sedentary behavior exposure questionnaire examined daily time spent on the following behavior through 5 items: reading (books, newspapers and magazines), writing, drawing and toy cars, puppets, and board games. The total sedentary behaviors were quantified as sedentary time (hours/day by the following formula: (typical weekday time*5 + typical weekend daytime*2)/7. Daily sedentary time ≥ 2 h was defined as a disapproving quantity.

Sleep duration was assessed by a self-reported question: “How many hours do you usually sleep each day, including daytime and night-time?” According to the sleep duration recommendation by the National Sleep Foundation of USA [23], < 8 h/day was defined as insufficient sleep duration.

The following social-demographic variables were collected: age, residence location, education level, income, currently smoking (Yes or No), alcohol drinking (Yes or No). Age groups were divided into 15–34 years versus 35–44 years; residence location was divided into urban versus rural; education level was divided into non/primary school, junior school, senior school, and college and above; income level was divided into tertiles: low, middle, and high.

### Statistical analyses

For descriptive analysis, means and SD (standard deviation) were used to describe continuous variables, and frequency and percentages for categorical variables. One-way ANOVA test, Kruskal-Wallis test and Chisquare test were used to examine group differences in social-demographics, nutrients intake, daily behaviors and obesity indices over time. Independent t-test and Chisquare test were used to compare the differences in nutrients intake and daily behaviors between the participants of central obesity vs. normal. Generalized linear mixed model was further performed to estimate the associations of nutrients intake and daily behaviors with central obesity. Effect size was expressed as OR and 95% CI (confidence intervals). Adjusted Model 1 was controlled for location, survey year, education level, income, currently smoking, and alcohol drinking; Adjusted Model 2, based on model 1, simultaneously controlled for nutrients intake, sleep duration, sedentary time, and LTPA. All data analysis was performed by SPSS (version 24.0), *P* < 0.05 (two tailed) was considered as statistically significant.

## Results

### Secular trends of central obesity, nutrients intake, and daily behaviors from 2004 to 2011

Table 1 shows the changes in central obesity, nutrients intake, and daily behaviors among Chinese women of childbearing age during the period from 2004 to 2011. The average WC increased from (75.9 ± 8.8) cm to (78.4 ± 9.6) cm, and the prevalence of high WC and high WHtR correspondingly increased, especially high WHtR, rose from 22.8% to 2004 to 32.6% in 2011 (all *p* values < 0.001). For nutrients intake, the proportion of carbohydrate intake above AMDR decreased over time, from 33.8 to 17.8%; contrastingly, the proportion of fats and proteins intake above AMDR increased significantly, from 41.1 to 56.4% and 14.5–25.3% respectively (all *p* values *<* 0.001). At the same time, sleep duration showed a significant downward trend, from (8.3 ± 1.1) hours in 2004 to (8.0 ± 1.0) hours in 2011, while sedentary time showed an increasing trend, from (2.8 ± 2.0) hours to (3.3 ± 2.2) hours (both *p <* 0.001).

**Table 1:** The characteristics of study population by survey year (CHNS 2004–2011)

Survey year	2004	2006	2009	2011	*F/χ²*	*P*
*Social demographics*						
Age (years)						
Mean ± SD	32.8 ± 7.3	34.3 ± 7.6	34.6 ± 7.4	35.6 ± 7.0	145.46	< 0.001
15–34	880 (50.7%)	846 (43.8%)	677 (40.2%)	469 (36.1%)	73.41	< 0.001
35–44	855 (49.3%)	1087 (56.2%)	1008 (59.8%)	831 (63.9%)		
Residence location						
Urban	587 (33.8%)	659 (34.1%)	558 (33.1%)	433 (33.3%)	0.48	0.924
Rural	1148 (66.2%)	1274 (65.9%)	1127 (66.9%)	867 (66.7%)		
Education level						
Non/primary school	508 (29.5%)	505 (26.4%)	465 (28.2%)	346 (26.9%)	81.10	< 0.001
Junior school	778 (45.2%)	856 (44.7%)	718 (43.5%)	552 (42.9%)		
Senior school	364 (21.2%)	430 (22.5%)	335 (20.3%)	233 (18.1%)		
College and above	70 (4.1%)	122 (6.4%)	131 (7.9%)	156 (12.1%)		
Income						
Low	898 (52.5%)	929 (48.9%)	497 (30.1%)	321 (25.3%)	493.08	< 0.001
Medium	589 (34.4%)	663 (34.9%)	664 (40.2%)	463 (36.4%)		
High	224 (13.1%)	306 (16.1%)	489 (29.6%)	487 (38.3%)		
Currently smoking						
Yes	27 (1.6%)	20 (1.0%)	16 (0.9%)	11 (0.8%)	4.48	0.216
No	1708 (98.4%)	1913 (99.0%)	1669 (99.1%)	1289 (99.2%)		
Alcohol drinking						
Yes	142 (8.4%)	154 (8.1%)	154 (9.4%)	132 (10.3%)	5.38	0.146
No	1542 (91.6%)	1739 (91.9%)	1481 (90.6%)	1150 (89.7%)		
*Nutrients intake*						
Carbohydrate (g)	305.0 ± 100.3	291.8 ± 106.1	271.9 ± 91.3	254.5 ± 95.9	235.46	< 0.001
Carbohydrate (%E)						
= AMDR	789 (46.2%)	867 (46.3%)	812 (49.9%)	595 (47.0%)	164.97	< 0.001
< AMDR	343 (20.1%)	467 (24.9%)	474 (29.1%)	445 (35.2%)		
> AMDR	577 (33.8%)	538 (28.7%)	341 (21%)	225 (17.8%)		
Fat (g)	65.0 ± 35.8	66.5 ± 35.2	70.0 ± 45.4	68.0 ± 37.5	5.02	0.002
Fat (%E)						
= AMDR	590 (34.5%)	562 (30.0%)	503 (30.9%)	367 (29.0%)	107.33	< 0.001
< AMDR	417 (24.4%)	413 (22.1%)	253 (15.6%)	185 (14.6%)		
> AMDR	702 (41.1%)	897 (47.9%)	871 (53.5%)	713 (56.4%)		
Protein (g)	63.8 ± 25.1	61.8 ± 20.7	62.3 ± 20.3	61.5 ± 21.3	7.39	0.061
Protein (%E)						
= AMDR	1155 (67.6%)	1289 (68.9%)	1084 (66.6%)	811 (64.1%)	102.78	< 0.001
< AMDR	307 (18.0%)	311 (16.6%)	213 (13.1%)	134 (10.6%)		
> AMDR	247 (14.5%)	272 (14.5%)	330 (20.3%)	320 (25.3%)		
*Daily behaviors*						
LTPA						
Yes	172 (9.9%)	188 (9.7%)	199 (11.8%)	128 (9.8%)	5.40	0.145
No	1563 (90.1%)	1745 (90.3%)	1486 (88.2%)	1172 (90.2%)		
Sedentary time (h/day)						
Mean ± SD	2.8 ± 2.0	2.8 ± 1.9	3.1 ± 2.3	3.3 ± 2.2	69.96	< 0.001
< 2 h	635 (36.9%)	636 (33.2%)	454 (27.6%)	316 (24.6%)	66.01	< 0.001
≥ 2 h	1087 (63.1%)	1282 (66.8%)	1193 (72.4%)	971 (75.4%)		
Sleep duration (h/day)						
Mean ± SD	8.3 ± 1.1	8.2 ± 1.0	8.1 ± 1.0	8.0 ± 1.0	57.39	< 0.001
≥ 8 h	1397 (83.0%)	1517 (80.9%)	1277 (78.6%)	977 (75.2%)	23.94	< 0.001
< 8 h	286 (17.0%)	357 (19.1%)	347 (21.4%)	305 (23.5%)		
*Central obesity indices*						
WC (cm)						
Mean ± SD	75.9 ± 8.8	76.3 ± 8.8	77.2 ± 9.4	78.4 ± 9.6	60.11	< 0.001
Normal	1360 (78.4%)	1502 (77.7%)	1264 (75.0%)	901 (69.3%)	40.10	< 0.001
High	375 (21.6%)	431 (22.3%)	421 (25.0%)	399 (30.7%)		
WHtR						
Mean ± SD	0.5 ± 0.1	0.5 ± 0.1	0.5 ± 0.1	0.5 ± 0.1	46.28	< 0.001
Normal	1339 (77.2%)	1492 (77.2%)	1247 (74.0%)	876 (67.4%)	48.69	< 0.001
High	396 (22.8%)	441 (22.8%)	438 (26.0%)	424 (32.6%)		

Data given as mean ± SD (standard deviation) or number (percent)

*%E* the percentage of energy intake from carbohydrate, fat, protein respectively, *AMDR* acceptable macronutrient distribution range, *LTPA* leisure time physical activity, *WC* waist circumference, *WHtR* waist to height ratio

### Comparisons of nutrients intake and daily behaviors by participants with central obesity versus normal groups

Table 2 shows the differences in nutrients intake and daily behaviors by central obesity vs. normal groups. Carbohydrate intake in women with central obesity were significantly higher than that of normal, no matter whether WC or WHtR was applied as indicators of central obesity (both *p* < 0.05). However, protein intake was only higher in high WC women (*p* = 0.001). The proportions of participation in LTPA were significantly lower among people with central obesity regardless of which indicator of central obesity was used (*p <* 0.001 for both). In addition, the high WHtR population had longer sleep duration (8.2 ± 1.0 h vs. 8.1 ± 1.0 h, *p* = 0.029) but shorter sedentary time (2.8 ± 2.0 h vs. 3.0 ± 2.1 h, *p* = 0.001); and people with high WC had shorter sedentary time (2.8 ± 1.9 h vs. 3.0 ± 2.2 h, *p* < 0.001) too. Table S1 shows the differences in sample characteristics between groups (see Additional file 1).

**Table 2:** Comparisons of nutrients intake and daily behaviors by normal groups vs. central obesity

	WC		*t/χ²*	*P*	WHtR		*t/χ²*	*P*
	Normal	High			Normal	High		
*Nutrients intake*								
Carbohydrate (g)	281.0 ± 98.6	289.0 ± 106.6	− 2.65	0.008	280.6 ± 98.3	289.9 ± 107.2	− 3.10	0.002
Carbohydrate (%E)								
= AMDR	2289 (74.7%)	774 (25.3%)	1.57	0.456	2264 (73.9%)	799 (26.1%)	2.34	0.311
< AMDR	1319 (76.3%)	410 (23.7%)			1308 (75.7%)	421 (24.3%)		
> AMDR	1273 (75.7%)	408 (24.3%)			1237 (73.6%)	444 (26.4%)		
Fat (g)	67.1 ± 38.0	67.9 ± 40.6	− 0.75	0.456	67.3 ± 38.3	67.2 ± 39.7	0.14	0.890
Fat (%E)								
= AMDR	1512 (74.8%)	510 (25.2%)	0.64	0.728	1493 (73.8%)	529 (26.2%)	0.36	0.835
< AMDR	961 (75.8%)	307 (24.2%)			942 (74.3%)	326 (25.7%)		
> AMDR	2408 (75.7%)	775 (24.3%)			2374 (74.6%)	809 (25.4%)		
Protein (g)	61.9 ± 21.6	64.0 ± 23.0	− 3.39	0.001	62.1 ± 21.8	63.1 ± 22.6	− 1.58	0.114
Protein (%E)								
= AMDR	3270 (75.4%)	1069 (24.6%)	1.87	0.392	3232 (74.5%)	1107 (25.5%)	0.76	0.683
< AMDR	742 (76.9%)	223 (23.1%)			706 (73.2%)	259 (26.8%)		
> AMDR	869 (74.3%)	300 (25.7%)			871 (74.5%)	298 (25.5%)		
*Daily behaviors*								
LTPA								
Yes	594 (86.5%)	93 (13.5%)	49.32	< 0.001	588 (85.6%)	99 (14.4%)	49.88	< 0.001
No	4433 (74.3%)	1533 (25.7%)			4366 (73.2%)	1600 (26.8%)		
Sedentary time (h/day)								
Mean ± SD	3.0 ± 2.2	2.8 ± 1.9	3.78	< 0.001	3.0 ± 2.1	2.8 ± 2.0	3.36	0.001
< 2 h	1543 (75.6%)	498 (24.4%)	0.13	0.716	1495 (73.2%)	546 (26.8%)	1.40	0.236
≥ 2 h	3408 (75.2%)	1125 (24.8%)			3383 (74.6%)	1150 (25.4%)		
Sleep duration (h/day)								
Mean ± SD	8.1 ± 1.0	8.2 ± 1.1	− 6.06	0.544	8.1 ± 1.0	8.2 ± 1.0	− 2.18	0.029
≥ 8 h	3896 (75.4%)	1272 (24.6%)	0.50	0.480	3818 (73.9%)	1350 (26.1%)	0.57	0.451
< 8 h	964 (74.4%)	331 (25.6%)			970 (74.9%)	325 (25.1%)		

Data given as mean ± SD (standard deviation) or number (percent)

*%E* the percentage of energy intake from carbohydrate, fat, protein respectively, *AMDR* acceptable macronutrient distribution range, *LTPA* leisure time physical activity, *WC* waist circumference, *WHtR* waist to height ratio

### Associations of nutrients intake and daily behaviors with central obesity

The association of nutrients intake and daily behaviors with central obesity is demonstrated in Tables 3, 4 and 5, where WC, WHtR and both of them are applied as indicators of central obesity respectively. As shown in Table 3, the crude regression models of generalized linear mixed analyses show that protein intake above the AMDR (OR = 1.20, 95% CI 1.05–1.37, *p* < 0.01) and non-participation in LTPA (OR = 1.82, 95% CI 1.48–2.23, *p* < 0.001) were risk factors for high WC. After adjusting for location, survey year, education level, income, currently smoking and alcohol drinking, the two factors still exerted a statistically significant effect (Adjusted Model 1). Nutrients intake, sleep duration, sedentary time, LTPA were further adjusted in Adjusted Model 2, the results kept stable, protein intake above the AMDR (OR = 1.21, 95% CI 1.05–1.39, *p* < 0.01) and non-participation in LTPA (OR = 1.45, 95% CI 1.17–1.80, *p* < 0.001) were still associated with high WC.

**Table 3:** Associations of nutrients intake and daily behaviors with central obesity (high WC)

	Crude model	Adjusted model1	Adjusted model2
*Carbohydrate intake (%E)*			
< AMDR vs. = AMDR	0.98 (0.87–1.12)	0.98 (0.86–1.12)	0.96 (0.82–1.12)
> AMDR vs. = AMDR	0.91 (0.80–1.04)	0.94 (0.82–1.08)	0.99 (0.80–1.23)
*Fat intake (%E)*			
< AMDR vs. = AMDR	0.95 (0.81–1.10)	0.96 (0.82–1.12)	0.97 (0.78–1.20)
> AMDR vs. = AMDR	1.01 (0.89–1.14)	1.00 (0.89–1.13)	1.04 (0.89–1.21)
*Protein intake (%E)*			
< AMDR vs. = AMDR	0.92 (0.79–1.07)	0.94 (0.81–1.10)	0.94 (0.80–1.10)
> AMDR vs. = AMDR	1.20 (1.05–1.37)**	1.19 (1.04–1.37)*	1.21 (1.05–1.39)**
*LTPA*			
No vs. Yes	1.82 (1.48–2.23)***	1.45 (1.17–1.79)***	1.45 (1.17–1.80)***
*Sedentary time*			
≥ 2 h vs. < 2 h	1.06 (0.94–1.18)	1.05 (0.93–1.18)	1.06 (0.94–1.19)
*Sleep duration*			
< 8 h vs. ≥ 8 h	1.04 (0.91–1.18)	1.01 (0.88–1.15)	1.02 (0.89–1.16)

*WC* waist circumference, *%E* the percentage of energy intake from carbohydrate, fat, protein respectively, *AMDR* acceptable macronutrient distribution range, *LTPA* leisure time physical activity, *Adjusted Model1* adjusted for location, survey year, education level, income, currently smoking and alcohol drinking, *Adjusted Model2* adjusted basing on Model 1 and carbohydrate intake, fat intake, protein intake, LTPA, sedentary time, sleep duration

*P < 0.05, **P < 0.01, ***P < 0.001

**Table 4:** Associations of nutrients intake, daily behaviors with central obesity (high WHtR)

	Crude model	Adjusted model1	Adjusted model2
*Carbohydrate intake (%E)*			
<AMDR vs. =AMDR	0.98 (0.86–1.12)	0.99 (0.86–1.13)	0.93 (0.79–1.09)
>AMDR vs. =AMDR	0.96 (0.85–1.10)	0.97 (0.85–1.11)	1.13 (0.92–1.40)
*Fat intake (%E)*			
<AMDR vs. =AMDR	0.94 (0.80–1.09)	0.95 (0.81–1.10)	0.87 (0.70–1.07)
>AMDR vs. =AMDR	1.02 (0.90–1.15)	1.03 (0.91–1.17)	1.11 (0.96–1.30)
*Protein intake (%E)*			
<AMDR vs. =AMDR	1.03 (0.89–1.20)	1.04 (0.89–1.21)	1.02 (0.88–1.19)
>AMDR vs. =AMDR	1.10 (0.96–1.26)	1.09 (0.95–1.26)	1.14 (0.98–1.32)
*LTPA*			
No vs. Yes	1.77 (1.46–2.16)***	1.39 (1.13–1.70)**	1.36 (1.10–1.67)**
*Sedentary time*			
≥ 2 h vs.<2 h	0.98 (0.88–1.10)	0.97 (0.87–1.09)	0.97 (0.86–1.09)
*Sleep duration*			
< 8 h vs. ≥8 h	0.96 (0.84–1.09)	0.93 (0.81–1.06)	0.94 (0.82–1.08)

*WHtR* weight to height ratio, *%E* the percentage of energy intake from carbohydrate, fat, protein respectively, *AMDR* acceptable macronutrient distribution range, *LTPA* leisure time physical activity, *Adjusted Model1* adjusted for location, survey year, education level, income, currently smoking and alcohol drinking, *Adjusted Model2* adjusted basing on Model 1 and carbohydrate intake, fat intake, protein intake, LTPA, sedentary time, sleep duration

*P < 0.05, **P < 0.01, ***P < 0.001

**Table 5:** Associations of nutrients intake, daily behaviors with central obesity (high WC and high WHtR)

	Crude model	Adjusted model1	Adjusted model2
*Carbohydrate intake (%E)*			
<AMDR vs. =AMDR	0.95 (0.83–1.10)	0.98 (0.85–1.13)	0.91 (0.77–1.08)
>AMDR vs. =AMDR	0.99 (0.86–1.13)	0.99 (0.86–1.14)	1.10 (0.88–1.37)
*Fat intake (%E)*			
<AMDR vs. =AMDR	1.05 (0.92–1.19)	1.00 (0.85–1.18)	0.94 (0.75–1.17)
>AMDR vs. =AMDR	0.98 (0.83–1.16)	1.04 (0.91–1.19)	1.13 (0.96–1.33)
*Protein intake (%E)*			
<AMDR vs. =AMDR	0.96 (0.81–1.13)	0.98 (0.83–1.15)	0.96 (0.81–1.14)
>AMDR vs. =AMDR	1.16 (1.01–1.33)*	1.15 (0.99–1.33)	1.19 (1.02–1.38)*
*LTPA*			
No vs. Yes	1.92 (1.54–2.40)***	1.53 (1.21–1.93)***	1.53 (1.21–1.94)***
*Sedentary time*			
≥ 2 h vs.<2 h	0.97 (0.87–1.10)	0.97 (0.85–1.09)	0.96 (0.85–1.09)
*Sleep duration*			
< 8 h vs. ≥8 h	0.99 (0.86–1.13)	0.95 (0.82–1.09)	0.96 (0.83–1.11)

*WC* waist circumference, *WHtR* weight to height ratio, *%E* the percentage of energy intake from carbohydrate, fat, protein respectively, *AMDR* acceptable macronutrient distribution range, *LTPA* leisure time physical activity, *Adjusted Model1* adjusted for location, survey year, education level, income, currently smoking and alcohol drinking, *Adjusted Model2* adjusted basing on Model 1 and carbohydrate intake, fat intake, protein intake, LTPA, sedentary time, sleep duration

*P < 0.05, **P < 0.01, ***P < 0.001

The crude regression model of generalized linear mixed analyses in Table 4 shows that non-participation in LTPA was an independent risk factor for high WHtR (OR = 1.77, 95% CI 1.46–2.16, *p <* 0.001). After two-step adjustment in Adjusted Model 1 (OR = 1.39, 95% CI 1.13–1.70, *p <* 0.01) and Adjusted Model 2 (OR = 1.36, 95% CI 1.10–1.67, *p <* 0.01), the effects were kept, however, attenuated. The association of nutrients intake, sleep duration, and sedentary behavior with high WHtR were not statistically significant.

Table 5 examined the associations of intake and daily behaviors with the combination of high WC and high WHtR. It can be seen that both protein intake above the AMDR (OR = 1.16, 95% CI 1.01–1.33, *p <* 0.05) and non-participation in LTPA (OR = 1.92, 95% CI 1.54–2.40, *p <* 0.001) were related factors. After taking fully adjustment (Adjusted model 2), they kept significant (OR = 1.19, 95% CI 1.02–1.38, *p <* 0.05; and OR = 1.53, 95% CI 1.21–1.94, *p <* 0.001, respectively); and the associations were stronger than in Tables 3 and 4, especially for non-participation in LTPA.

All above analyses were repeated by age-stratified, where it was further found that non-participated in LTPA was an independent influencer for central obesity in younger (15–34 years old) women of childbearing age, while in the older age group (35–44 years old), protein intake above the AMDR was a major contributor to central obesity (*p* < 0.05 for all above) (Table S3**–**S5 for 15–34 age group, Table S6–S8 for 35–44 age group, in Additional file 1).

## Discussion

In recent decades, increasing researches suggested a strong link between obesity and reproduction health [24], which indicated that obesity in women of childbearing age should be in great concern. Our findings revealed an increasing track of central obesity among women of childbearing age over time, the prevalence of high WC increased from 21.6 to 30.7% and so did the prevalence of high WHtR from 22.8 to 32.6%. In the same period, diets and behaviors changed dramatically, including less carbohydrate, more fat and protein intake, lower participation rates in physical activity, longer sedentary time and shorter sleep duration. Among which, lack of physical activity and too much protein intake were found to be contributors of central obesity. Our findings provide evidence that Chinese women of childbearing age are facing challenge of increasing central obesity and unhealthy lifestyle may be involved in the uptrend.

Compared to overweight/obesity, central obesity is more sensitive in predicting health outcomes. A previous study among polycystic ovary syndrome (PCOS) patients confirmed that women with central obesity rather than obesity tended to have higher insulin resistance, lipid profiles and ovarian dysfunction [25]. Most existing studies observe the cross-sectional prevalence of overweight/obesity by using BMI as indicator among women of childbearing age. A recent study conducted in China focused on the long-time trend of central obesity among women of childbearing age, revealing an increased track from 2004 (30.4%) to 2015 (45.5%) [4], which was a similar to our finding. However, the study applied the uniform cutoff value of central obesity without considering the natural growth of WC with age [19, 20], which made the prevalence of central obesity was slightly higher than our data, nevertheless, they all confirmed that the central obesity rate among women of childbearing age has been increasing.

Diet construction was usually considered to be important for the prevention of obesity [26]. Our study indicated that the construction of energy supply has certainly changed among women of childbearing age. They tended to intake less carbohydrates but more fats and proteins from their daily diets, while excessive protein intake was a major contributor for central obesity. These changes indicated that the continuing socio-economic development has gradually westernized the diet of Chinese women of childbearing age, enabling them to eat more meat, eggs, milk and other protein-rich foods. The effects of protein on obesity vary from their resources. A Belgian study showed that compared with animal protein, the intake of plant protein was negatively correlated with women’s BMI and waist circumference [27], and to the contrary, high animal protein intake, especially red meat, has been linked to weight gain. These results suggest that the composition of protein sources needs to be optimized when the proportion of protein in the diet of Chinese women of childbearing age has increased. The present study did not find the association between fat intake and central obesity among women of childbearing age. Generally, the correlation between fat intake and obesity has been controversial [28, 29], which may be due to different types of fat intake, obesity standards, and ways of body measurement and intake estimation; physiological variances in different groups should also be considered. Further investigations are needed to evaluate the impact of fat intake on central obesity aiming at this special population.

Unhealthy daily behaviors are also key factors for central obesity. In this study, a significantly shortened of sleep duration and increased of sedentary time were observed, whereas the participation rate of LTPA did not appear a growth trend, which suggested that the daily behaviors of Chinese women of childbearing age were gradually becoming unhealthy. We found that lack of LTPA was a prior contributor to both high WC and high WHtR, but sleep duration and sedentary time did not contribute to either of them. The inverse effect of lack of physical activity on central obesity has been widely demonstrated [30, 31]; however, there is no solid evidence whether sedentary time resulted in central obesity [32, 33]. Nevertheless, some studies have emphasized that sedentary behavior has the greatest impact on the health of people without exercise habits, which means that exercise habits can partially counteract the harmful effects of sitting too much [34, 35]. Therefore, from a public health standpoint, it is indispensable to encourage women of childbearing age to reduce sedentary time, given the high prevalence of non-participation in LTPA (approximately 90%) in this study. Our study also showed that short sleep duration did not associate with high WC and high WHtR of women. Some studies [36, 37] support our finding, but more representative and standardized studies are needed to consolidate this conclusion. Therefore, it is necessary to develop effective physical activity promotion measures for Chinese women of reproductive age, as well as to reduce sedentary time and increase sleep duration, as these factors are strongly associated with central obesity in women of reproductive age.

Additionally, age difference was found in the lead contributors for central obesity in women of childbearing age. To the best of our knowledge, this is the first time such a difference has been found and may be worth further investigation. An animal experiment elucidated that for the mice with a high-energy diet feed, estrogen could reduce lipid deposition in skeletal muscle to strengthen muscle and improve insulin resistance [38], that is to say estrogen may help to resist the effects of overnutrition on obesity for the mice; but population-based evidence is very limited. Therefore, we speculated that decline of estrogen levels with older age may account for why protein intake above the AMDR contributed to the central obesity in older age group instead of the younger group, and future studies are warranted to confirm this age difference. Nevertheless, it reminds us that different measures could be taken for the obesity control in childbearing-age women of different age, for example, encourage younger women to do exercise instead of controlling diet solely, while older age women should pay more attention to the structure and quality of their diet.

To the best of our knowledge, this is the first study to report the trend of central obesity, along with nutrients intake and daily behaviors, and their associations among women of childbearing age in China. The study was based on a large sample and longitudinal data, and the representativeness of CHNS respondents ensures the generalizability of the research conclusions to an extent. In addition, the cut-off value of central obesity was divided according to age, so as to avoid the mixed influence of age on waist circumference. Nevertheless, there are several limitations in our study. Firstly, the 3-day diet survey may not be representative enough for the dietary habits of the respondents. Further, the information about LTPA, sleep duration and sedentary time were all based on self-report of the respondents, therefore report bias were inevitable. Moreover, since the CHNS survey defined leisure time physical activity for only six types of common sports, other sports and exercise manner, such as commuting, work, and housework, were not included, which may lead to an overestimation of the percentage of people who did not exercise, as well as the intensity of LTPA was failed to be measured either. Additionally, other variables, such as chronic diseases history and drug use, may involve in the associations, however, they were unavailable in the study.

## Conclusions

From 2004 to 2011, the prevalence of central obesity in Chinese women of childbearing age increased from 21.6 to 30.7% (WC as indice), and 22.8–32.6% (WHtR as indice). Dietary and physical activity patterns have changed over the same period, particularly a higher contribute rate of protein in total energy and consistently low rates of LTPA participation, both of which could be implicated with the increasing central obesity in women of childbearing age. Furthermore, for the younger women, did not do LTPA was the lead factor for central obesity, while for the older women, the main influencer was the higher proportion of protein account for energy intake. Our findings would be of significant in making targeted prevention and control strategy to respond to the increasing central obesity issue in women of childbearing age.

## Supplementary Information

**Additional file 1. Table S1**. Differences of social-demographics by normal groups vs. central obesity;** Table S2**. The characteristics of women who participated in survey for 4 waves;** Table S3–S8**. Associations of nutrients intake and daily behaviors with central obesity by age stratified.

## Data Availability

The datasets analyzed during the current study are available from the website: https://www.cpc.unc.edu/projects/china/data/datasets.

## Abbreviations

WC: Waist circumference
WHtR: Waist to height ratio
AMDR: Acceptable macronutrient distribution range
LTPA: Leisure time physical activity
