# Supplementary material for: Trends of central obesity and associations with nutrients intake and daily behaviors among women of childbearing age in China
Source: BMC Womens Health. 2022 Jan 12;22:12. doi: 10.1186/s12905-022-01600-9 (PMC8753840; doi:10.1186/s12905-022-01600-9)
Supplement: Supplementary file 1 — Additional file 1. Table S1. Differences of social-demographics by normal groups vs. central obesity; Table S2. The characteristics of women who participated in survey for 4 waves; Table S3–S8. Associations of nutrients intake and daily behaviors with central obesity by age stratified. [file 12905_2022_1600_MOESM1_ESM.doc]

| **Table S1. Differences of social-demographics by normal groups vs. central obesity (CHNS 2004-2011)** | | | | | | | | | |
| --- | --- | --- | --- | --- | --- | --- | --- | --- | --- |
|  | **WC** | | ***t/χ²*** | ***P*** | **WHtR** | | | ***t/χ²*** | ***P*** |
|  | **Normal** | **High** |  |  | **Normal** | | **High** |  |  |
| **Social demographics** | |  |  |  |  | |  |  |  |
| Age(years) |  |  |  |  | |  |  |  |  |
| mean ± SD | 33.8±7.6 | 35.5±6.4 | -9.05 | <0.001 | | 33.8±7.7 | 35.4±6.4 | -8.69 | <0.001 |
| 15-34 | 2198 (76.5%) | 674 (23.5%) | 2.59 | 0.108 | | 2151 (74.9%) | 721 (25.1%) | 0.50 | 0.480 |
| 35-44 | 2829 (74.8%) | 952 (25.2%) |  |  | | 2803 (74.1%) | 978 (25.9%) |  |  |
| Residence location | |  |  |  | |  |  |  |  |
| urban | 1750 (78.2%) | 487 (21.8%) | 13.01 | <0.001 | | 1731 (77.4%) | 506 (22.6%) | 15.09 | <0.001 |
| rural | 3277 (74.2%) | 1139 (25.8%) |  |  | | 3223(73.0%) | 1193 (27.0%) |  |  |
| Education level |  |  |  |  | |  |  |  |  |
| non/primary  school | 1286 (70.5%) | 538 (29.5%) | 56.19 | <0.001 | | 1212 (66.4%) | 612 (33.6%) | 118.37 | <0.001 |
| junior school | 2172 (74.8%) | 732 (25.2%) |  |  | | 2155 (74.2%) | 749 (25.8%) |  |  |
| senior school | 1089 (80.0%) | 273 (20.0%) |  |  | | 1097 (80.5%) | 265 (19.5%) |  |  |
| college and above | 400 (83.5%) | 79 (16.5%) |  |  | | 410 (85.6%) | 69 (14.4%) |  |  |
| Income |  |  |  |  | |  |  |  |  |
| low | 1998 (75.5%) | 647 (24.5%) | 0.24 | 0.887 | | 1950 (73.7%) | 695 (26.3%) | 2.69 | 0.260 |
| medium | 1807 (76.0%) | 572 (24.0%) |  |  | | 1772 (74.5%) | 607 (25.5%) |  |  |
| high | 1134 (75.3%) | 372 (24.7%) |  |  | | 1145 (76.0%) | 361 (24.0%) |  |  |
| Currently smoking |  |  |  |  | |  |  |  |  |
| Yes | 51 (68.9%) | 23 (31.1%) | 1.79 | 0.181 | | 52 (70.3%) | 22 (29.7%) | 0.69 | 0.406 |
| No | 4976 (75.6%) | 1603 (24.4%) |  |  | | 4902 (74.5%) | 1677 (25.5%) |  |  |
| Alcohol drinking |  |  |  |  | |  |  |  |  |
| Yes | 443 (76.1%) | 139 (23.9%) | 0.28 | 0.595 | | 436 (74.9%) | 146 (25.1%) | 0.21 | 0.644 |
| No | 4441 (75.1%) | 1471 (24.9%) |  |  | | 4377 (74.0%) | 1535 (26.0%) |  |  |
| Data given as mean ± SD (standard deviation) or number (percent). WC: waist circumference; WHtR: waist to height ratio. | | | | | | | | | |

| **Table S2. The characteristics of women who participated in survey for 4 waves (n=534)** | | | | | | |
| --- | --- | --- | --- | --- | --- | --- |
| **Survey year** | **2004** | **2006** | **2009** | **2011** | ***F/x²*** | ***P*** |
| **Age (mean ± SD)** | 31.7±4.7 | 33.6±4.7 | 36.7±4.7 | 38.6±4.7 | 226.023 | <0.001 |
| **Nutrients intake** |  |  |  |  |  |  |
| Carbohydrate (g) | 305.3±94.7 | 295.0±102.3 | 280.6±91.1 | 261.8±97.9 | 19.965 | <0.001 |
| Carbohydrate (%E) |  |  |  |  |  |  |
| =AMDR | 263(50.0%) | 251(47.9%) | 247(47.2%) | 240(46.2%) | 47.198 | <0.001 |
| <AMDR | 94(17.9%) | 116(22.1%) | 150(28.7%) | 174(33.5%) |  |  |
| >AMDR | 169(32.1%) | 157(30.0%) | 126(24.1%) | 106(20.4%) |  |  |
| Fat (g) | 63.6±33.8 | 65.8±34.3 | 72.6±64.3 | 69.0±40.1 | 4.018 | 0.007 |
| Fat (%E) |  |  |  |  |  |  |
| =AMDR | 182(34.6%) | 166(31.7%) | 156(29.8%) | 154(29.6%) | 27.411 | <0.001 |
| <AMDR | 130(24.7%) | 113(21.6%) | 92(17.6%) | 83(16.0%) |  |  |
| >AMDR | 214(40.7%) | 245(46.8%) | 275(52.6%) | 283(54.4%) |  |  |
| Protein (g) | 62.9±25.4 | 61.1±20.2 | 62.1±19.8 | 62.0±22.0 | 0.625 | 0.599 |
| Protein (%E) |  |  |  |  |  |  |
| =AMDR | 368(70.0%) | 360(68.7%) | 354(67.7%) | 336(64.6%) | 36.851 | <0.001 |
| <AMDR | 90(17.1%) | 95(18.1%) | 77(14.7%) | 58(11.2%) |  |  |
| >AMDR | 68(12.9%) | 69(13.2%) | 92(17.6%) | 126(24.2%) |  |  |
| **Daily behaviors** |  |  |  |  |  |  |
| LTPA |  |  |  |  |  |  |
| Yes | 34(6.4%) | 35(6.6%) | 38(7.1%) | 46(8.6%) | 2.499 | 0.475 |
| No | 500(93.6%) | 499(93.4%) | 496(92.9%) | 488(91.4%) |  |  |
| Sedentary time (h/day) |  |  |  |  |  |  |
| mean ± SD | 2.6±1.7 | 2.6±1.9 | 3.0±2.1 | 3.2±1.9 | 8.898 | <0.001 |
| <2h | 196(37.0%) | 176(33.1%) | 150(28.2%） | 125(23.5%) | 25.853 | <0.001 |
| ≥2h | 334(63.0%) | 355(66.9%) | 381(71.8%) | 407(76.5%) |  |  |
| Sleep duration (h/day) |  |  |  |  |  |  |
| mean ± SD | 8.3±1.0 | 8.2±1.0 | 8.0±1.0 | 8.0±1.0 | 14.029 | <0.001 |
| ≥8h | 441(84.5%) | 432(83.1%) | 413(78.7%) | 390(73.4%) | 24.349 | <0.001 |
| <8h | 81(15.5%) | 88(16.9%) | 112(21.3%) | 141(26.6%） | |  |
| **Central obesity indices** | |  |  |  |  |  |
| WC (cm) |  |  |  |  |  |  |
| mean ± SD | 75.3±8.6 | 76.9±8.4 | 78.0±9.5 | 79.4±9.3 | 22.955 | <0.001 |
| Normal | 412(77.2%) | 421(78.8%) | 388(72.7%) | 360(67.4%) | 21.799 | <0.001 |
| High | 122(22.8%) | 113(21.2%) | 146(27.3%) | 174(32.6%) |  |  |
| WHtR |  |  |  |  |  |  |
| mean ± SD | 0.5 ± 0.1 | 0.5 ± 0.1 | 0.5 ± 0.1 | 0.5 ± 0.1 | 20.232 | <0.001 |
| Normal | 398(74.5%) | 426(79.8%) | 385(72.1%) | 355(66.5%) | 24.889 | <0.001 |
| High | 136(25.5%) | 108(20.2%） | 149(27.9%) | 179(33.5%) |  |  |
| Data given as mean ± SD (standard deviation) or number (percent). | | | | | | |
| %E: the percentage of energy intake from carbohydrate, fat, protein respectively; AMDR: acceptable macronutrient distribution | | | | | | |
| range; LTPA: leisure time physical activity; WC: waist circumference; WHtR: waist to height ratio. | | | | | | |

| **Table S3. Associations of nutrients intake and daily behaviors with central obesity (high WC) of 15-34 age group** | | | |
| --- | --- | --- | --- |
|  | **Crude Model** | **Adjusted Model1** | **Adjusted Model2** |
| **Carbohydrate intake (%E)** |  |  |  |
| <AMDR vs. =AMDR | 0.96(0.77-1.18) | 0.91(0.71-1.17) | 0.91(0.71-1.18) |
| >AMDR vs. =AMDR | 1.03(0.84-1.27) | 1.00(0.70-1.42) | 1.07(0.74-1.53) |
| **Fat intake (%E)** |  |  |  |
| <AMDR vs. =AMDR | 1.14(0.90-1.45) | 1.19(0.83-1.71) | 1.14(0.79-1.64) |
| >AMDR vs. =AMDR | 1.05(0.87-1.28) | 1.15(0.90-1.46) | 1.17(0.92-1.50) |
| **Protein intake (%E)** |  |  |  |
| <AMDR vs. =AMDR | 0.88(0.69-1.12) | 0.94(0.73-1.20) | 0.93(0.72-1.19) |
| >AMDR vs. =AMDR | 1.10(0.90-1.35) | 1.09(0.88-1.36) | 1.12(0.90-1.39) |
| **LTPA** |  |  |  |
| No vs. Yes | 2.24(1.69-2.97) *** | 1.80(1.34-2.42) *** | 1.82(1.35-2.46) *** |
| **Sedentary time** |  |  |  |
| ≥2h vs.<2h | 0.97(0.81-1.17) | 1.04(0.86-1.27) | 1.05(0.86-1.28) |
| **Sleep duration** |  |  |  |
| <8h vs. ≥8h | 0.99(0.80-1.23) | 1.00(0.80-1.25) | 1.02(0.81-1.27) |

**P* < 0.05, ***P* < 0.01, ****P* < 0.001.

WC: waist circumference; %E: the percentage of energy intake from carbohydrate, fat, protein respectively;

AMDR: acceptable macronutrient distribution range; LTPA: leisure time physical activity;

Adjusted Model1: Adjusted for location, survey year, education level, income, currently smoking and alcohol drinking;

Adjusted Model2: Adjusted basing on Model 1 and carbohydrate intake, fat intake, protein intake, LTPA, sedentary time, sleep duration.

| **Table S4. Associations of nutrients intake and daily behaviors with central obesity (high WHtR) of 15-34 age group** | | | |
| --- | --- | --- | --- |
|  | **Crude Model** | **Adjusted Model1** | **Adjusted Model2** |
| **Carbohydrate intake (%E)** |  |  |  |
| <AMDR vs. =AMDR | 0.89(0.72-1.08) | 0.79(0.62-1.01) | 0.81(0.63-1.03) |
| >AMDR vs. =AMDR | 0.97(0.79-1.19) | 1.19(0.84-1.67) | 1.27(0.90-1.80) |
| **Fat intake (%E)** |  |  |  |
| <AMDR vs. =AMDR | 0.96(0.76-1.21) | 0.88(0.63-1.24) | 0.83(0.59-1.17) |
| >AMDR vs. =AMDR | 1.03(0.85-1.25) | 1.26(1.00-1.60) | 1.29(0.99-1.65) |
| **Protein intake (%E)** |  |  |  |
| <AMDR vs. =AMDR | 1.08(0.86-1.37) | 1.07(0.84-1.35) | 1.06(0.83-1.35) |
| >AMDR vs. =AMDR | 1.02(0.83-1.25) | 1.05(0.84-1.30) | 1.07(0.85-1.33) |
| **LTPA** |  |  |  |
| No vs. Yes | 1.97(1.51-2.57) *** | 1.50(1.13-2.00) ** | 1.53(1.15-2.04) ** |
| **Sedentary time** |  |  |  |
| ≥2h vs.<2h | 0.99(0.82-1.18) | 1.02(0.85-1.24) | 1.02(0.84-1.24) |
| **Sleep duration** |  |  |  |
| <8h vs. ≥8h | 0.80(0.64-1.01) | 0.80(0.63-1.01) | 0.81(0.64-1.03) |

**P* < 0.05, ***P* < 0.01, ****P* < 0.001.

WHtR: weight to height ratio; %E: the percentage of energy intake from carbohydrate, fat, protein respectively;

AMDR: acceptable macronutrient distribution range; LTPA: leisure time physical activity;

Adjusted Model1: Adjusted for location, survey year, education level, income, currently smoking and alcohol drinking;

Adjusted Model2: Adjusted basing on Model 1 and carbohydrate intake, fat intake, protein intake, LTPA, sedentary time, sleep duration.

| **Table S5. Associations of nutrients intake and daily behaviors with central obesity (high WC&high WHtR) of 15-34 age group** | | | |
| --- | --- | --- | --- |
|  | **Crude Model** | **Adjusted Model1** | **Adjusted Model2** |
| **Carbohydrate intake (%E)** |  |  |  |
| <AMDR vs. =AMDR | 0.90(0.72-1.13) | 0.82(0.63-1.06) | 0.82(0.63-1.07) |
| >AMDR vs. =AMDR | 1.00(0.80-1.24) | 1.03(0.70-1.50) | 1.09(0.74-1.60) |
| **Fat intake (%E)** |  |  |  |
| <AMDR vs. =AMDR | 1.10(0.86-1.42) | 1.17(0.80-1.70) | 1.10(0.75-1.61) |
| >AMDR vs. =AMDR | 1.05(0.85-1.30) | 1.22(0.95-1.58) | 1.25(0.96-1.63) |
| **Protein intake (%E)** |  |  |  |
| <AMDR vs. =AMDR | 0.92(0.71-1.18) | 0.96(0.74-1.24) | 0.95(0.73-1.23) |
| >AMDR vs. =AMDR | 1.07(0.86-1.32) | 1.06(0.84-1.34) | 1.07(0.85-1.36) |
| **LTPA** |  |  |  |
| No vs. Yes | 2.25(1.65-3.07) *** | 1.78(1.28-2.49) ** | 1.81(1.29-2.55) ** |
| **Sedentary time** |  |  |  |
| ≥2h vs.<2h | 0.93(0.77-1.12) | 0.98(0.80-1.20) | 0.98(0.80-1.20) |
| **Sleep duration** |  |  |  |
| <8h vs. ≥8h | 0.95(0.75-1.20) | 0.94(0.74-1.21) | 0.95(0.74-1.22) |

**P* < 0.05, ***P* < 0.01, ****P* < 0.001.

WC: waist circumference; WHtR: weight to height ratio;

%E: the percentage of energy intake from carbohydrate, fat, protein respectively;

AMDR: acceptable macronutrient distribution range; LTPA: leisure time physical activity;

Adjusted Model1: Adjusted for location, survey year, education level, income, currently smoking and alcohol drinking;

Adjusted Model2: Adjusted basing on Model 1 and carbohydrate intake, fat intake, protein intake, LTPA, sedentary time, sleep duration.

| **Table S6. Associations of nutrients intake and daily behaviors with central obesity (high WC) of 35-44 age group** | | | |
| --- | --- | --- | --- |
|  | **Crude Model** | **Adjusted Model1** | **Adjusted Model2** |
| **Carbohydrate intake (%E)** |  |  |  |
| <AMDR vs. =AMDR | 0.86(0.72-1.02) | 0.98(0.80-1.19) | 0.86(0.72-1.02) |
| >AMDR vs. =AMDR | 0.99(0.85-1.17) | 1.03(0.80-1.34) | 0.99(0.85-1.17) |
| **Fat intake (%E)** |  |  |  |
| <AMDR vs. =AMDR | 0.83(0.68-1.01) | 0.79(0.61-1.04) | 0.83(0.68-1.01) |
| >AMDR vs. =AMDR | 0.97(0.83-1.13) | 0.96(0.79-1.17) | 0.97(0.83-1.13) |
| **Protein intake (%E)** |  |  |  |
| <AMDR vs. =AMDR | 0.95(0.78-1.16) | 0.95(0.77-1.16) | 0.95(0.78-1.16) |
| >AMDR vs. =AMDR | 1.28(1.07-1.52) * | 1.28(1.06-1.53) * | 1.29(1.07-1.56) ** |
| **LTPA** |  |  |  |
| No vs. Yes | 1.34(1.01-1.80) | 1.08(0.79-1.48) | 1.34(1.04-1.80) |
| **Sedentary time** |  |  |  |
| ≥2h vs.<2h | 1.11(0.96-1.28) | 1.06(0.91-1.23) | 1.11(0.96-1.28) |
| **Sleep duration** |  |  |  |
| <8h vs. ≥8h | 1.08(0.92-1.26) | 1.06(0.90-1.24) | 1.08(0.92-1.26) |

**P* < 0.05, ***P* < 0.01, ****P* < 0.001.

WC: waist circumference; %E: the percentage of energy intake from carbohydrate, fat, protein respectively;

AMDR: acceptable macronutrient distribution range; LTPA: leisure time physical activity;

Adjusted Model1: Adjusted for location, survey year, education level, income, currently smoking and alcohol drinking;

Adjusted Model2: Adjusted basing on Model 1 and carbohydrate intake, fat intake, protein intake, LTPA, sedentary time, sleep duration.

| **Table S7. Associations of nutrients intake and daily behaviors with central obesity (high WHtR) of 35-44 age group** | | | |
| --- | --- | --- | --- |
|  | **Crude Model** | **Adjusted Model1** | **Adjusted Model2** |
| **Carbohydrate intake (%E)** |  |  |  |
| <AMDR vs. =AMDR | 1.06(0.90-1.25) | 1.03(0.84-1.26) | 1.02(0.82-1.25) |
| >AMDR vs. =AMDR | 0.98(0.83-1.17) | 1.13(0.88-1.46) | 1.12(0.86-1.45) |
| **Fat intake (%E)** |  |  |  |
| <AMDR vs. =AMDR | 0.95(0.77-1.16) | 0.85(0.65-1.12) | 0.86(0.66-1.13) |
| >AMDR vs. =AMDR | 1.02(0.87-1.19) | 1.02(0.83-1.25) | 1.02(0.84-1.26) |
| **Protein intake (%E)** |  |  |  |
| <AMDR vs. =AMDR | 1.01(0.83-1.23) | 1.00(0.82-1.22) | 0.99(0.81-1.21) |
| >AMDR vs. =AMDR | 1.17(0.97-1.39) | 1.18(0.97-1.43) | 1.20(0.99-1.47) |
| **LTPA** |  |  |  |
| No vs. Yes | 1.59(1.18-2.14) ** | 1.24(0.90-1.70) | 1.23(0.89-1.68) |
| **Sedentary time** |  |  |  |
| ≥2h vs.<2h | 0.99(0.85-1.14) | 0.93(0.80-1.09) | 0.94(0.80-1.10) |
| **Sleep duration** |  |  |  |
| <8h vs. ≥8h | 1.05(0.90-1.24) | 1.03(0.87-1.22) | 1.04(0.87-1.24) |

**P* < 0.05, ***P* < 0.01, ****P* < 0.001.

WHtR: weight to height ratio; %E: the percentage of energy intake from carbohydrate, fat, protein respectively;

AMDR: acceptable macronutrient distribution range; LTPA: leisure time physical activity;

Adjusted Model1: Adjusted for location, survey year, education level, income, currently smoking and alcohol drinking;

Adjusted Model2: Adjusted basing on Model 1 and carbohydrate intake, fat intake, protein intake, LTPA, sedentary time, sleep duration.

| **Table S8. Associations of nutrients intake and daily behaviors with central obesity (high WC&high WHtR) of 35-44 age group** | | | |
| --- | --- | --- | --- |
|  | **Crude Model** | **Adjusted Model1** | **Adjusted Model2** |
| **Carbohydrate intake (%E)** |  |  |  |
| <AMDR vs. =AMDR | 1.05(0.88-1.25) | 0.98(0.79-1.21) | 0.96(0.77-1.20) |
| >AMDR vs. =AMDR | 0.94(0.79-1.13) | 1.19(0.91-1.56) | 1.16(0.88-1.52) |
| **Fat intake (%E)** |  |  |  |
| <AMDR vs. =AMDR | 0.90(0.73-1.12) | 0.78(0.59-1.03) | 0.80(0.61-1.06) |
| >AMDR vs. =AMDR | 1.03(0.88-1.22) | 1.06(0.86-1.31) | 1.06(0.86-1.32) |
| **Protein intake (%E)** |  |  |  |
| <AMDR vs. =AMDR | 0.99(0.80-1.23) | 0.98(0.79-1.22) | 0.98(0.79-1.21) |
| >AMDR vs. =AMDR | 1.23(1.02-1.49) * | 1.27(1.03-1.56) * | 1.29(1.04-1.58) * |
| **LTPA** |  |  |  |
| No vs. Yes | 1.61(1.15-2.25) ** | 1.29(0.91-1.84) | 1.28(0.90-1.82) |
| **Sedentary time** |  |  |  |
| ≥2h vs.<2h | 1.01(0.87-1.18) | 0.95(0.81-1.12) | 0.96(0.81-1.12) |
| **Sleep duration** |  |  |  |
| <8h vs. ≥8h | 1.02(0.86-1.20) | 0.99(0.83-1.18) | 1.00(0.83-1.19) |

**P* < 0.05, ***P* < 0.01, ****P* < 0.001.

WC: waist circumference; WHtR: weight to height ratio;

%E: the percentage of energy intake from carbohydrate, fat, protein respectively;

AMDR: acceptable macronutrient distribution range; LTPA: leisure time physical activity;

Adjusted Model1: Adjusted for location, survey year, education level, income, currently smoking and alcohol drinking;

Adjusted Model2: Adjusted basing on Model 1 and carbohydrate intake, fat intake, protein intake, LTPA, sedentary time, sleep duration.
